# Caveolin-1 reduces HIV-1 infectivity by restoration of HIV Nef mediated impairment of cholesterol efflux by apoA-I

**DOI:** 10.1186/1742-4690-9-85

**Published:** 2012-10-15

**Authors:** Shanshan Lin, Peter E Nadeau, Xiaomei Wang, Ayalew Mergia

**Affiliations:** 1 Department of Infectious Disease and Pathology, University of Florida, Gainesville, Florida, 32611, USA

**Keywords:** HIV, Caveolin-1, Cholesterol efflux, Nef, Apolipoprotein A-I

## Abstract

**Background:**

HIV infection results in inhibited cholesterol efflux by apolipoprotein A-I (apoA-I) in macrophages, and this impairment involves Nef mediated down-regulation and redistribution of ATP-binding cassette transporter A1 (ABCA-1). We investigated the effect of caveolin-1 (Cav-1) on the cholesterol efflux by apoA-I in HIV infected primary and THP-1 cell-differentiated macrophages as well as astrocyte derived glioblastoma U87 cells.

**Results:**

Our results reveal that Cav-1 restores the Nef -mediated impairment of cholesterol efflux by apoA-I in both cell types. Co-immunoprecipitation studies indicate a physical association of Cav-1 and Nef. The level of ABCA-1 expression remains the same whether Cav-1 is over-expressed or not. In addition, we examined the cholesterol composition of HIV particles released from Cav-1 treated cells and identified that the cholesterol content is dramatically reduced. The infectivity level of these virus particles is also significantly decreased.

**Conclusions:**

These observations suggest that the interplay of Cav-1 with Nef and cholesterol subsequently counters Nef induced impairment of cholesterol efflux by apoA-l. The findings provide a cellular mechanism by which Cav-1 has an ability to restore HIV mediated impairment of cholesterol efflux in macrophages. This subsequently influences the cholesterol content incorporated into virus particles thereby inhibiting HIV infectivity and contributing to HIV’s persistent infection of macrophages.

## Background

Caveolin 1 (Cav-1), a 21~24-kDa scaffolding protein, is an important structural component of caveolae [1], small invaginations of the plasma membrane, which are enriched in cholesterol, phospholipids, and sphingolipids. This protein is highly expressed in terminally differentiated cells including endothelial cells, macrophages, dendritic cells and adipocytes [2,3]. Functional studies have shown that Cav-1 is involved in a wide range of cellular processes, including cell cycle regulation, signal transduction, endocytosis, cholesterol trafficking and efflux [3-9]. Multiple lines of evidence indicate that Cav-1 acts as a scaffolding protein capable of directly interacting with and modulating the activity of caveolin-bound signaling molecules. The Cav-1 scaffolding domain (CSD), residues 82 to 101, is essential for both Cav-1 oligomerization and the interaction of caveolin with other proteins [10]. Associations with other proteins through the CSD help provide coordinated and efficient signal transduction [11,12]. The CSD serves as a receptor for binding proteins containing the sequence φXφXXXXφ, φXXXXφXXφ, or φXφXXXXφXXφ (φ representing any aromatic amino acid and X any other amino acid)[10]. HIV Env has been shown to interact with Cav-1 via a motif (WNNMTWMQW) localized within the ectodomain (the C-terminal heptad repeats) of HIV-1 gp41 [13-15]. Our group has shown the binding of Cav-1 with HIV Env in the lipid rafts which subsequently blocks cell fusion and innocent bystander killing mediated by HIV envelope [16]. We have also demonstrated that HIV infection in primary human monocyte derived macrophages (MDMs) results in a dramatic up-regulation of Cav-1 expression mediated by HIV Tat [17]. Furthermore, over-expression of Cav-1 causes significant reduction in HIV replication in macrophages. Cav-1 inhibits HIV replication through transcriptional repression of viral gene expression by modulating the NF-κB pathway [18]. The up-regulation of Cav-1 by HIV infection and subsequent inhibition of HIV replication suggest a role for Cav-1 in macrophage persistent infection.

Cav-1 plays an important role in cellular cholesterol homeostasis, a process that controls intracellular lipid composition and prevents cholesterol accumulation. Cav-1 has been implicated in modulating the expression of lipoprotein receptors and interacts with many lipid transporter molecules [11,19-21]. Furthermore, it is involved in the transport of newly synthesized cholesterol from the endoplasmic reticulum (ER) to the plasma membrane [11,22,23] and promotes cholesterol efflux in hepatic cells [9,24]. HIV appears to manipulate cellular cholesterol metabolism to ensure that there is a sufficient supply of cholesterol and that it is located in the appropriate compartments such as lipid rafts for efficient virus release and subsequent infectivity [25-28]. Cholesterol is an important component that influences HIV production and efficient virus infectivity. Cholesterol depletion significantly reduces HIV-1 particle production [29-34]. Virus infectivity is also negatively affected when HIV is produced from cholesterol depleted cells [26,35].

The HIV accessory protein Nef has an ability to exploit cholesterol metabolism. Proposed mechanisms for this strategy include binding to cholesterol and aiding the transport of newly synthesized cholesterol into lipid rafts and viral particles as well as enhancing cholesterol synthesis [36,37]. Nef has also been shown to impair ATP binding cassette transporter protein 1 (ABCA-1)-dependent cholesterol efflux from human macrophages by down-regulation and redistribution of ABCA-1 [38]. This suggests that Nef is involved in HIV mediated cholesterol accumulation. Since Cav-1 has a high affinity for cholesterol and aids in the transport of newly synthesized cholesterol from the ER to the plasma membrane and indirectly promoting the transfer to extracellular acceptors such as lipid free apolipoprotein A-I (apoA-I) we hypothesize it would influence the level of cholesterol accumulation as well as virus production and infectivity. Macrophages are major targets for HIV infection and also play an important role in its pathogenesis. The up-regulation of Cav-1 by HIV infection and the role of Cav-1 in cholesterol trafficking suggest a mechanism for a Cav-1/cholesterol mediated impact on HIV replication in macrophages. In this report, we establish evidence for a Cav-1/cholesterol mediated mechanism of inhibition of HIV replication for the first time providing a new angle in understanding HIV’s persistent infection of macrophages.

## Results

### Cav-1 restores HIV Nef mediated impairment of cholesterol efflux by apoA-I in U87 cells and macrophages

**Figure 1:**
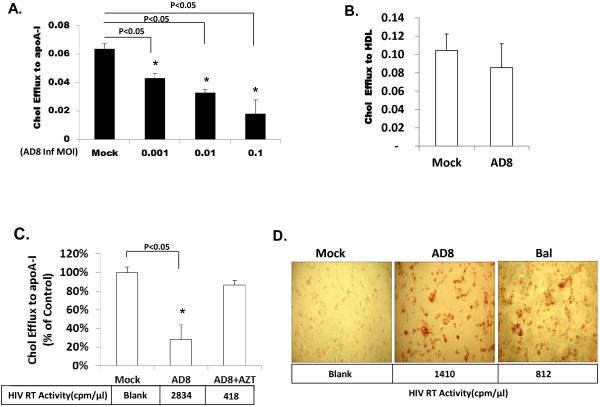
**HIV-1 impairs apoA-I mediated cholesterol efflux from THP-1 cell-differentiated macrophages.** (**A**) HIV AD8 infected and uninfected THP-1 cell-differentiated macrophages were cultured for 14 days. MOI represents multiplicity of infection. Cells were labeled with [^3^H] cholesterol, and subsequently incubated with media in the presence and absence of apoA-I (50 μg/ml). ApoA-I-induced cholesterol efflux was measured and analyzed as described in the Materials and Methods. (**B**) Cells were treated the same as above but incubated with medium in the presence or in absence of HDL (50 μg/ml) and HDL mediated cholesterol efflux was measured. (**C**) ApoA-I mediated cholesterol efflux was determined in the presence and absence of the HIV replication inhibitor AZT (5uM). All experiments were performed in triplicate, and results shown are mean ± SD with P values. (**D**) Uninfected or infected with HIV AD8 (moi 0.01) and HIV Bal (moi 0.001) THP-1 cell-differentiated macrophages cultured for 10 days then incubated with AcLDL (50μg/ml) for 48 h followed by 30μg/ml apoA-I stimulation for 18 hours. Lipid accumulation was determined by Oil red O staining by light microscopy. HIV reverse transcriptase activity in culture medium is shown in the bottom panel.

HIV infection impairs ATP-binding cassette transporter A1 (ABCA-1) dependent cholesterol efflux by apoA-l. The Nef protein is identified as the key molecule responsible for this effect [38]. Since Cav-1 is an important regulator of cholesterol trafficking and is involved in the transport of newly synthesized cholesterol from the ER to the plasma membrane, it is likely to influence Nef mediated ABCA-1 dependent down modulation of cholesterol efflux. To determine whether Cav-1 counters the influence of Nef on cholesterol trafficking, first, we tested the impairment of cholesterol efflux in HIV infected THP-1 cell-differentiated macrophages. HIV AD8 infected THP-1 cells were exposed to lipid-free apoA-I or HDL treatment to induce cholesterol efflux. Cholesterol efflux was measured as the fraction of total radiolabeled cholesterol appearing in the medium in the presence of apoA-I after subtraction of values for apoA-I-free medium [39]. ApoA-I stimulated cholesterol efflux from HIV infected THP-1 cell-differentiated macrophages was markedly decreased in a dose dependent manner with the reduction reaching 71.6% as compared to uninfected cells (Figure 1A). No significant difference was observed between HIV infected and uninfected cells in HDL mediated cholesterol efflux (Figure 1B). The decrease in cholesterol efflux to apoA-I by HIV infection was not present in the presence of AZT, an inhibitor of the HIV replication (Figure 1C). These results suggest that HIV infection decreased the apoA-l mediated cholesterol efflux substantially and are in accordance with previous findings [38]. To further examine impairment of cholesterol efflux due to HIV infection the level of cholesterol accumulation was tested by oil red O staining of infected macrophages. As shown in Figure 1D, accumulated cholesterol was markedly increased in AD8 or Bal infected macrophages as compared to uninfected cells which is similar to previous findings [38]. 

**Figure 2:**
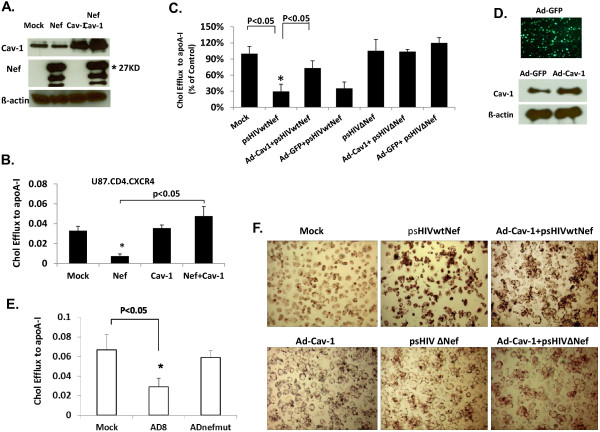
**Cav-1 restores HIV −1 Nef mediated impairment of cholesterol efflux to apoA-I.** U87-CD4-CXCR4 cells were transfected with Nef expression plasmid (pcDNA3.1SF2Nef) along with pCZ-cav-1 or pCZ vector. Cells were labeled with [^3^H] cholesterol for 36 h. (**A**) The level of Nef and Cav-1 expression was determined by Western blot analysis. (**B**) ApoA-I induced cholesterol efflux is shown. All experiments were performed in triplicate, and results shown are mean ± SD with P values. (**C**) Primary monocyte derived macrophages (MDMs) were infected with Ad-Cav-1 or Ad-GFP, followed by infection with VSV-G pseudotyped HIV, either carrying Nef (psHIVwtNef) or defective Nef (psHIVΔNef) at an moi of 5. ApoA-I mediated cholesterol efflux was performed and analyzed by incubating cells in medium in the presence or absence of 50 μg/ml apoA-I. The results are presented as a percentage of cholesterol efflux to apoA-I from control (set as 100%), and are the mean± SD of triplicate determinations. The expression levels of Cav-1 and GFP are shown in (**D**). (**E**) Primary macrophages were infected with AD8 or nef defective AD8 (ADnefmut) at an moi of 0.01. ApoA-I mediated cholesterol efflux was measured 15 days post infection. (**F**) THP-1 cell-differentiated macrophages were infected with psHIVwtNef or psHIVΔNef at an moi 3 with or without Ad-Cav-1. On day 5 after infection, Oil red O staining was performed and a representative area in each well is shown.

To address the influence of Cav-1 on cholesterol efflux of HIV infected cells, we examined whether Cav-1 can restore Nef mediated impairment of cholesterol efflux. First, U87-CD4-CXCR4 cells were transfected with a Cav-1 expression construct (pCZ-Cav-1) in the presence or absence of a Nef expression plasmid (pcDNA3.1SF2Nef). ApoA-l or HDL mediated cholesterol efflux was measured by harvesting the culture media as well as cell lysate samples. As a control U87-CD4-CXCR4 cells were also transfected with expression vector lacking Cav-1 (pCZ-vector) or Nef (pcDNA3.1). The expression of Cav-1 and Nef in transfected cells was determined by Western blot analysis (Figure 2A). As expected apoA-l mediated cholesterol efflux from cells transfected with the Nef expression construct alone was reduced by 77% compared to cells transfected with the plasmid construct devoid of nef or cells that received pCZ-Cav-1 (Figure 2B). Interestingly, apoA-l mediated cholesterol efflux from cells co-transfected with pCZ-Cav-1 and pcDNA3.1SF2Nef was comparable, and even slightly higher, to that of mock which did not receive Nef treatment, suggesting that Cav-1 can restore the impairment of cholesterol efflux caused by Nef. Neither Nef nor Cav-1 had significant effect on HDL mediated cholesterol efflux (data not shown). We confirmed our findings by conducting these studies in physiologically relevant primary monocyte derived macrophages (MDMs). We infected MDMs with vesicular stomatitis virus glycoprotein (VSV-G) Env pseudotyped pSG3^Δenv^ HIV provirus carrying wild type nef (psHIVwtNef) or defective nef (psHIVΔNef) for one round of replication. Infection of MDMs with psHIVwtNef showed a significant reduction (70%) in apoA-l mediated cholesterol efflux as compared to uninfected cells (Figure 2C) similar to what is observed in the U87-CD4-CXCR4 cells (Figure 2B). The reduction in cholesterol efflux was 75% when compared to Nef defective HIV. There was no significant difference in cholesterol efflux between Nef defective HIV infected and uninfected MDMs. Introduction of exogenous Cav-1 into psHIVwtNef MDMs using adenovirus expressing Cav-1 (Ad-Cav-1) increased cholesterol efflux by 43% compared to cells only infected with psHIVwtNef. The control adenovirus carrying GFP (Ad-GFP) was not able to counter cholesterol efflux impairment induced by psHIVwtNef infection of the MDMs (Figure 2C). As expected, Western blot analysis of MDMs transduced with Ad-GFP revealed endogenous Cav-1 expression, with increased amounts of Cav-1 in MDMs treated with Ad-Cav-1 (Figure 2D). Furthermore, introduction of exogenous Cav-1 or GFP using adenovirus did not alter cholesterol efflux from MDMs infected with the Nef defective HIV. In addition, we compared apoA-I mediated cholesterol efflux in MDMs infected with wild type AD8 and replication competent nef deleted AD8 (ADnefmut) viruses. Cholesterol efflux was decreased by 56.3% in wild type infected macrophages compared to uninfected cells while ADnefmut infection had no significant effect (Figure 2E). These results taken together suggest that Cav-1 is capable of restoring HIV induced impairment of apoA-1 mediated cholesterol efflux. To confirm our findings, we further examined the affect of Cav-1 on cholesterol accumulation by oil red O staining in HIV infected THP-1 cell-differentiated macrophage cells. As shown in Figure 2F, psHIVwtNef infected cells had significantly increased cholesterol accumulation compared to uninfected cells (mock) which is similar to HIV AD8 infected THP-1 cell-differentiated macrophages (Figure 1D). The co-infection of macrophages with Ad-Cav-1 and psHIVwtNef, on the other hand, showed a dramatic reduction of intracellular cholesterol inclusions when compared to psHIVwtNef only infected cells. As expected, no significant influence on intracellular cholesterol accumulation was observed when THP-1 cell-differentiated macrophages were infected with Nef defective HIV (psHIVΔNef). In addition, over-expression of Cav-1 by Ad-Cav-1 infection did not alter intracellular cholesterol inclusions in cells infected with psHIVΔNef.

**Figure 3:**
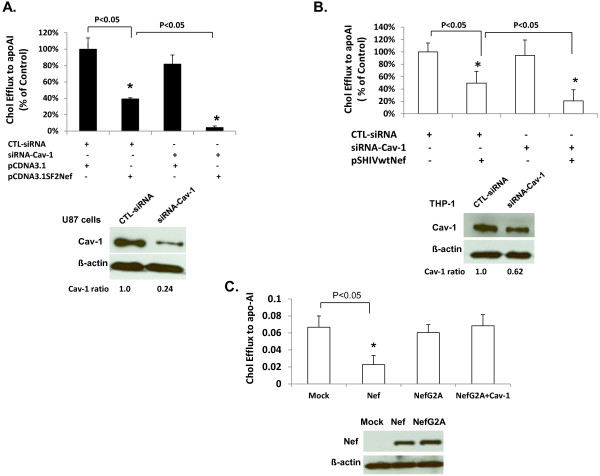
**siRNA knockdown of Cav-1 and its effect on cholesterol of cells expressing wild type Nef or NefG2A.** (**A**). U87-CD4-CXCR4 cells were transfected with Cav-1 siRNA or control siRNA (CTL-siRNA) followed by transfection with Nef expressing plasmid (pSHIVwtNef) or pCDNA3.1 vector. The level of Cav-1 expression in siRNA treated cells was detected by Western blotting analysis. Cells were labelled with [^3^H] cholesterol for 36 hours and apoA-I induced cholesterol efflux was measured. Results are shown as the percentage of cholesterol efflux relative to control. (**B**) THP-1 cell-differentiated macrophages were first transfected with Cav-1 siRNA or CTL-siRNA which was followed by infection with VSV-G pseudotyped HIV (psHIVwtNef) at an moi of 3. Cells were again treated with siRNA the day after infection. Cholestrol efflux was measuered as described above four days post-infection. (**C**) U87-CD4-CXCR4 cells were transfected with pCI (Mock), pCI NL4-3 Nef-HA-WT (Nef), pCI NL4-3 NefG2A-HA (NefG2A), or pCI NL4-3 NefG2A-HA along with pCZ-cav-1 (Nef plus Cav-1). Cholestrol efflux to apoA-I was determined. All experiments were performed in triplicate and results shown are mean ± SD with P values.

We further determined whether endogenous Cav-1 has an effect on Nef mediated suppression of cholesterol efflux to apoA-1 in U87 cells and THP-1 cell-differentiated macrophages. The expression of endogenous Cav-1 was knocked down using specific siRNA, and the expression of Nef was accomplished by transfecting U87 cells with pCDNA3.1SF2Nef or pseudotyped HIV (psHIVwtNef) infection of THP-1 cell-differentiated macrophages. The siRNA treatment reduced the expression of Cav-1 by 76% in U87 cells and 38% in THP-1 cell-differentiated macrophages (Figure 3A and 3B, respectively). Cholesterol efflux to apoA1 was reduced by 61% in Nef expressing U87 cells in the absence of siRNA targeting Cav-1 (Figure 3A). The level of cholesterol was further reduced (96%) when Cav-1 expression was knocked-down with Cav-1 specific siRNA. Similar results were observed in THP-1 cell-differentiated macrophages showing a decrease in cholesterol efflux by 50% in Nef expressing cells and by 79% in Cav-1 siRNA treated Nef expressing cells (Figure 3B). Furthermore, the levels of cholesterol efflux correlate with the efficiency of siRNA knock-down in U87 and THP-1 cells (Figure 3A and 3B).

In addition, to determine whether Cav-1 specifically restores Nef mediated impairment of cholesterol efflux to apoA-l, U87 cells were co-transfected with a Nef mutant (NefG2A) and Cav-1 expressing plasmids. The NefG2A is a Nef mutant that cannot undergo myristoylation [40]. Its association with the plasma membrane is impaired [26,35], and it lacks the ability to decrease apoA-l stimulated cholesterol efflux [36,41]. As shown in Figure 3C, cells expressing Nef experienced 62% less cholesterol efflux to apoA-I compared to Mock. In contrast, NefG2A had no effect on apoA-l mediated cholesterol efflux in the presence of either endogenous or over-expressing Cav-1 cells. These studies, therefore, clearly establish that Cav-1 counters Nef mediated impairment of cholesterol efflux by apoA-l.

### Cav-1 over-expression has no effect on ABCA-1 expression

**Figure 4:**
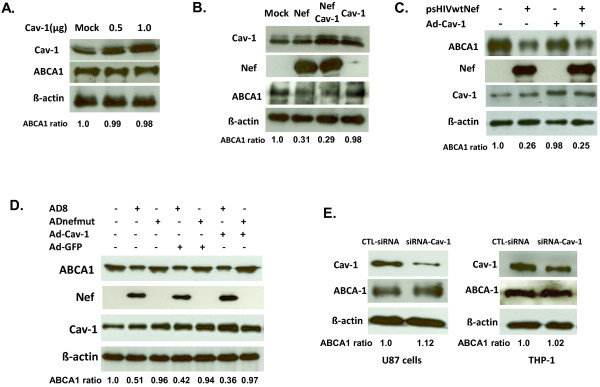
**Cav-1 over-expression has no influence on ABCA-1 expression.** (**A**) U87-CD4-CXCR4 cells were transiently transfected either with pCZ-vector (mock) or with different doses of Cav-1 (pCZ-Cav-1). Expression of ABCA-1 protein was examined by Western blot analysis. (**B**) Cells were transfected with pCZ-vector (mock), Nef expressing construct (pcDNA3.1SF2Nef) and pCZ-Cav-1 or pCZ-Cav-1 only. The expression levels of ABCA-1 in the presence or absence of Nef were determined in the cells with endogenous or over-expressing Cav-1. (**C**) MDMs were infected with VSV-G pseudotyped HIV (psHIVwtNef), Ad-Cav-1, or both. The level of Cav-1 and ABCA-1 expression was examined by Western blot analysis in the presence and absence of Cav-1 and/or Nef. Representative results from three experiments are shown in **A**, **B** and **C**. (**D**) MDMs were co-infected with wild type AD8 or nef defective AD8 (ADnefmut) and Ad-Cav-1 or Ad-GFP. The level of expression of ABCA-1, Nef, and Cav-1 was examined by Western blot analysis. (**E**) To determine whether siRNA knock-down of Cav-1 affects the expression of ABCA-1 samples used in experiment 3A and B were used to measure the level of ABCA-1 expression. Note that the siRNA Cav-1 knock out and β-actin are the same bands shown in Figure 3 because the same samples were used to demonstrate the level of ABCA-1 expression. The densities of bands corresponding to each protein were quantified using image densitometer analysis. The numbers at the bottom of each blot are the relative values of ABCA1 expression in transfected or transduced cells compared to those in control cells (mock).

Nef has been shown to impair ABCA1-dependent cholesterol efflux from human macrophages, and the expression of ABCA-1 is shown to be down regulated by HIV infection or Nef expression [38,42]. Cav-1 is implicated in positive regulation of ABCA-1 expression and ABCA-1 expression is down regulated in Cav-1 knockout mice [43]. In order to understand the mechanism responsible for Cav-1 mediated restoration of cholesterol efflux upon HIV infection we examined the expression of ABCA-1 in Cav-1 over-expressing cells. U87-CD4-CXCR4 cells were transfected with pCZ-Cav-1 in a dose-dependent manner, and the level of ABCA-1 expression was monitored by Western blot analysis. As shown in Figure 4A, Cav-1 over-expression did not alter the level of ABCA-1 expression. To further confirm our findings, we co-transfected U87 cells with pCZ-Cav-1 and pcDNA3.1SF2Nef, and then analyzed ABCA-1 expression by Western blot in the presence and absence of Cav-1 or Nef. Although Nef down-regulated ABCA-1 (by 69%) the level of ABCA-1 expression remained the same in Nef treated cells whether Cav-1 is over-expressed or not (Figure 4B), suggesting that Cav-1 mediated restoration of cholesterol efflux is not related to the regulation of ABCA-1 expression. Likewise, VSV-G pseudotyped HIV (psHIVwtNef) infection down-regulated ABCA-1 expression in MDMs, and co-infection of MDMs with psHIVwtNef and Ad-Cav-1 did not restore the reduced ABCA-1 levels (Figure 4C). MDMs were also infected with AD8 or ADnefmut virus along with infection of Ad-Cav-1 or Ad-GFP to determine the level of ABCA-1 expression. ADnefmut HIV infection did not affect the expression of ABCA-1 with either Ad-Cav-1 or Ad-GFP co-infection (Figure 4D). ABCA-1 expression, however, was decreased when MDMs were co-infected with AD8 and Ad-Cav-1 or Ad-GFP confirming the role of Nef in the reduction of ABCA-1 expression while over expression of Cav-1 has no impact on ABCA-1 expression. Furthermore, we examined the level of ABCA-1 expression in U87 or THP-1 cells where the expression of endogenous Cav-1 was knocked down with siRNA using samples described for results in Figure 3A and 3B. As shown in Figure 4E reduced endogenous Cav-1 expression by siRNA treatment did not alter the level of ABCA-1 expression. 

### Interaction of Nef and Cav-1

**Figure 5:**
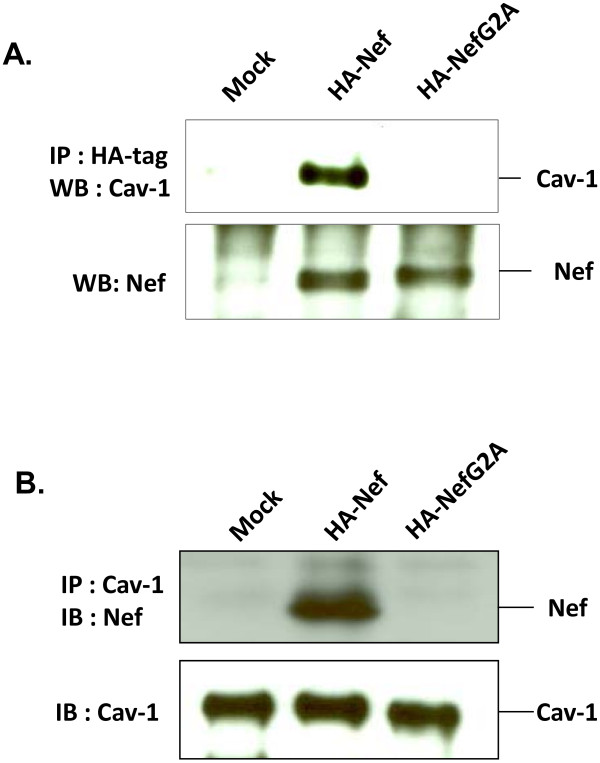
**Interaction of Cav-1 and Nef.** U87-CD4-CXCR4 cells were co-transfected with pCZ-Cav-1 and HA tagged Nef (pCI NL4-3 Nef-HA-WT) or NefG2A(pCI NL4-3 NefG2A-HA). Twenty-four hours post transfection the cells were cultured in the presence of cholesterol (30 μg/ml) for 48 hours followed by apoA-I (20 μg/ml) for 10 min. (**A**) The cells were harvested and subjected to immunoprecipitation using anti-HA antibody and immunoblots using anti-Cav-1 or anti-Nef antibody. (**B**) Alternatively, immunoprecipitaion was performed using anti-Cav-1 and immunoblotting using anti-Nef antibody.

Since over-expression of Cav-1 does not alter ABCA-1 expression, the mechanism of restoration of cholesterol efflux by Cav-1 that is impaired by Nef may not involve the level of ABCA-1 expression. Nef has been shown to bind to ABCA-1 [38,42], and it is not known whether Cav-1 interacts with Nef. Cav-1 may associate, either directly or indirectly, with Nef thereby countering the impairment of cholesterol efflux. To determine whether there is a physical association between Cav-1 and Nef, we performed co-immunoprecipitation and immunoblotting experiments. U87-CD4-CXCR4 cells were co-transfected with pCZ-Cav-1 and HA-tagged wild type Nef (pCI NL4-3 Nef-HA-WT) or the NefG2A mutant (pCI NL4-3 NefG2A-HA). Transfected cells were then cultured in medium containing cholesterol (30μg/ml) for 48 hours followed by treatment with apoA-I (20μg/ml) for 30min. Cell lysates were then subjected to co-immunoprecipitation and analyzed for Cav-1 and Nef interactions by immunoblotting. As shown in Figures 5A and 5B the association of Cav-1 and Nef is evident in U87 cells. Interestingly, there was no NefG2A mutant interaction with Cav-1 implicating the association of Nef and Cav-1 is at the cell membrane. We examined the endogenous interaction of Cav-1 and ABCA-1 in U87 cells and were unable to show ABCA-1 association with Cav-1 by co-immunopreciptation and immunoblotting analysis (data not shown). The interaction of Cav-1 with Nef suggests that Cav-1 by associating with Nef blocks the activity of Nef and subsequently helps restore cholesterol efflux impaired by Nef. 

### Cav-1 reduces HIV-1 infectivity by reducing the cholesterol content of virus particles

**Figure 6:**
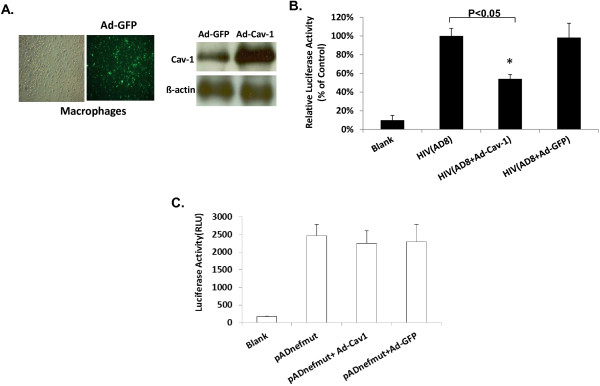
**Cav-1 over-expression inhibits HIV-1 infectivity.** MDMs were infected with AD8 or ADnefmut at an moi of 0.1 alone or in combination with Ad-Cav-1 or Ad-GFP. (**A**) The levels of GFP and Cav-1 expression are shown. (**B**) and (**C**) Infectious particles harvested from culture supernatants were titered and normalized. Level of infectivity was measured by infecting TZM-bl cells and subsequent luciferase assay. All experiments were performed in triplicate and results shown are mean ± SD with P values.

**Figure 7:**
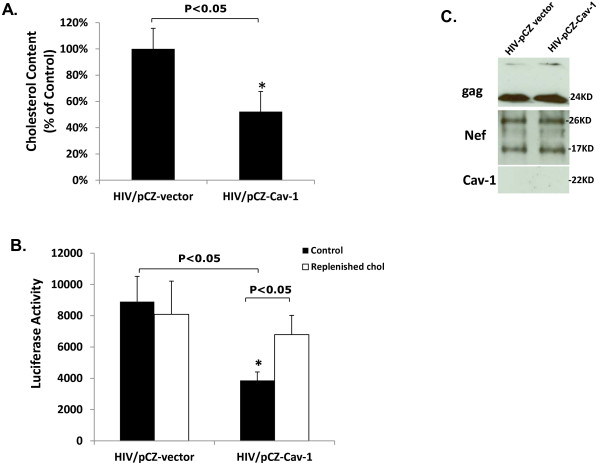
**Cav-1 over-expression reduces the cholesterol content in HIV-1 virus particles.** (**A**) Proviral DNA genome NL4-3 was transfected into U87 cells either with pCZ-vector or with pCZ-Cav-1. Virus particles generated were concentrated and normalized by p24 assay. Cholesterol contents were measured using the Amplex Red cholesterol Assay Kit. The results are presented as percentage of cholesterol content relative to control (HIV/pCZ-vector, set as 100%) and are the mean± SD of triplicate experiments with P values. (**B**) Normalized samples were replenished with exogenous cholesterol, and the level of infectivity was measured by infection of TZM-bl cells and luciferase assay. All experiments were performed in triplicate, and results shown are mean ± SD with P values. (**C**) Normalized samples from samples of (7**A**) were subjected to Western blot analysis using antibody to Gag, Nef, and Cav-1 to determine whether Cav-1 protein is incorporated in virus particles.

HIV is well known to rely on the host cellular cholesterol machinery for efficient replication and particle formation [25,26,28,30,44]. Since our results show that Cav-1 restores Nef impaired cholesterol efflux, we sought to determine if the promotion of this efflux by Cav-1 would have an impact on the infectivity of released virus particles. In order to demonstrate whether Cav-1 influences HIV infectivity, primary macrophages (MDMs) were transduced with Ad-Cav-1 or the control Ad-GFP. The level of GFP and Cav-1 expression in macrophages from which culture supernatant harvested is shown in Figure 6A. Twenty-four hours post transduction the macrophages were infected with HIV AD8. Virions produced from these infected macrophages were titered using the TZM-bl indicator cell line and normalized for the infectivity studies. As shown in Figure 6B infectivity of virus harvested from Cav-1 treated macrophages was reduced by 46% compared to Cav-1 untreated HIV infected cells. There was no significant difference in infectivity of virus particles obtained from Ad-GFP transduced cells when compared to that of virus harvested from Cav-1 untreated HIV infected cells. Similar experiments were performed using ADnefmut infections. Contrary to what was observed with AD8 HIV the level of infectivity of ADnefmut virus harvested from Ad-Cav-1 or Ad-GFP treated cells remained the same (Figure 6C). This, therefore, establishes that Cav-1 impairs HIV infectivity implicating that this may be linked to Cav-1 mediated promotion of cholesterol efflux by apoA-I that is impaired by Nef during HIV infection. Since Cholesterol within the HIV particle is strictly required for infection, our next set of experiments were aimed at investigating whether the reduction of HIV infectivity is related to the modulation of lipid content of the virions. HIV provirus DNA was co-transfected into U87 cells with pCZ-Cav-1 or pCZ-vector. Virus harvested from transfected cells was concentrated and normalized by a p24 ELISA assay. Equal amounts of virus particles were used to measure the cholesterol composition in the virion by the Amplex Red cholesterol Assay Kit. As shown in Figure 7A the cholesterol content of virus particles harvested from cells receiving Cav-1 was reduced by 48% compared to cells transfected with pCZ-vector. In addition, cholesterol was replenished within concentrated virus that was normalized and equal amounts were treated with (2-Hydroxypropyl)-ß-Cyclodextrin (CD) and saturated exogenous cholesterol to see if infectivity could be restored. Infectivity of cholesterol replenished virus was measured by luciferase activity in infected TZM-bl cells. The infectivity of virus particles collected on Cav-1 treated cells was reduced by 58% as compared to those collected on cells treated by pCZ-vector (Figure 7B). There was no difference in infectivity of cholesterol replenished and control viral particles collected from pCZ-vector treated cells (Figure 7B). As might be expected the infectivity of cholesterol replenished virus particles collected from Cav-1 treated cells was increased by 37% compared to the virus particles harvested from Cav-1 treated control (Figure 7B). In addition, we examined whether Cav-1 is incorporated into the virion to make sure that such incorporation has not affected infectivity directly rather than influencing the cholesterol content of the HIV virus particles. As shown in Figure 7C, we observed that Cav-1 protein is not incorporated in the virus particles as determined by Western blot analysis. Therefore, Cav-1 reduces virus infectivity by promoting cholesterol efflux which consequently decreased the availability of cholesterol during viral particle formation. 

## Discussion

HIV has been indicated to manipulate host cholesterol metabolism, leading to excessive cholesterol accumulation in infected T cells or macrophages [38,45], thereby supporting efficient viral replication. In the absence of proper esterification to fatty acid and efflux, cholesterol accumulates in the endoplasmic reticulum eventually leading to ER dysfunction and the activation of an ER stress associated apoptosis pathway [46-48]. Cav-1 is an important cellular cholesterol regulator, and its expression is dramatically enhanced in HIV infected macrophages [17], implicating a role for Cav-1 in HIV associated cholesterol alterations. Cav-1 is a structural component of Caveolae membrane microdomains, which have been suggested to play an important role in cholesterol trafficking and efflux. In this study, we investigate the effect of Cav-1 on the cholesterol efflux in HIV infected macrophages and human astrocytes-derived glioblastoma U87 cells. Our results show that Cav-1 restores the Nef induced impairment of cholesterol efflux by apoA-I. Furthermore, this restoration causes a reduction in the cholesterol composition of virus particles leading to decreased HIV infectivity. This suggests a role for Cav-1 in macrophage HIV persistent infection by enhancing cholesterol efflux.

Our results show neither Nef nor Cav-1 had significant effect on HDL mediated cholesterol efflux. HDL plays an important role in reverse cholesterol transport (RCT), in which HDL transports cholesterol from peripheral tissues to liver for excretion. RCT is a multifaceted, dynamic pathway which is involved with multiple molecules and effectors. The first step in RCT is ABCA-1 dependent efflux of cholesterol and phospholipids to apoA-I, the major component of HDL. ABCA-1 interacts with apoA-I and stimulates free cholesterol and phospholipids efflux responsible for nascent HDL formation [49]. Wang *et al.*[50] reported that ABCA-1 expression markedly increases apoA-I but not HDL mediated lipid efflux; the reason could be that compared with HDL, apoA-I is the preferred acceptor for ABCA1-promoted cholesterol and phospholipid efflux. We also found upon HIV infection Nef down regulates ABCA-1 expression, which dramatically inhibits apoA-I mediated cholesterol efflux, whereas HDL mediated cholesterol efflux was not affected by HIV infection. Over-expression of Cav-1 restores the impaired cholesterol efflux to apoA-I significantly, but not so much on intact HDL cholesterol efflux.

Promotion of cholesterol efflux by over-expression of Cav-1 is observed in hepatic cells [9]. Cav-1 can enhance the transfer of cholesterol to cholesterol-rich domains in the plasma membrane, where it is accessible to efflux. Multiple mechanisms are proposed for Cav-1’s regulation of cholesterol homeostasis. These include the modulation of the expression of lipoprotein receptors and the activity of proteins involved in lipid metabolism as well as interactions with lipid transport or transport of cholesterol to the plasma membrane facilitating cholesterol efflux [6-8,22,43]. ABCA-1 expression is important in regulating cholesterol efflux to apoA-I and it has been implicated that ABCA-1 stimulates the reorganization of plasma membrane microdomains to facilitate cholesterol efflux to apoA-I [51,52]. Cav-1 can regulate cholesterol homeostasis by modulating the expression of lipid regulators. Reduced levels of ABCA-1 have been observed in macrophages of Cav-1 knockout mice [43]. Our results show that we observe no change in the level of ABCA-1 expression when Cav-1 is over-expressed suggesting that the endogenous Cav-1 expression is sufficient enough to maintain physiologically relevant levels of ABCA-1 and that additional amounts of Cav-1 does not have an impact on ABCA-1 expression. The reduced level of ABCA-1 observed in the knockout mice is in complete absence of Cav-1 expression. ABCA-1 dependent cholesterol efflux can be impaired by HIV Nef mediated down modulation and altering of the intracellular distribution of ABCA-1 [38,42]. Similarly we observed a 69% decrease in ABCA-1 expression in the presence of Nef. Interestingly the decrease in ABCA-1 remains the same when additional amounts of Cav-1 are provided indicating that the reversal of Nef’s effect on cholesterol efflux by Cav-1 is not related to the level of ABCA-1 expression. Inhibition of ABCA-1 protein expression, as it pertains to Nef, in part depends upon the ER associated proteasomal degradation mechanism [42]. An unknown additional pathway unrelated to proteasomal activity is also suggested to contribute to ABCA-1 degradation. Although ABCA-1 is shown to interact with Nef the physical association is not essential for Nef mediated down-regulation of ABCA-1 efflux activity [38,42]. However, the influence of cellular distribution of ABCA-1 by Nef has been determined using confocal microscopy with Nef causing a prominent trapping of ABCA-1 in the ER [42]. ABCA-1 expression has been implicated in influencing the redistribution of cholesterol and Cav-1 [52]. Redistribution of Cav-1 from punctate caveolae-like structures to the general area of the plasma membrane is observed upon ABCA-1 expression. Our co-immunoprecipitation study reveals an interaction between Cav-1 and Nef. Furthermore, our observation that Cav-1 does not interact with the myristoylation defective Nef (NefG2A mutant) implicates an association of these proteins at the plasma membrane. These observations suggest that the interplay of Cav-1 with Nef and cholesterol subsequently counters Nef induced impairment of cholesterol efflux by apoA-l. In addition, since caveolae is a major source and platform for cholesterol efflux [4] over-expression of Cav-1 may induce the formation of more caveolae, which should subsequently enhance cholesterol efflux. The presence of Cav-1 in macrophages and its up-regulation upon HIV infection, therefore, can contribute to increased cholesterol efflux in these cells.

Cholesterol is an important structural component of HIV particles and their cholesterol content is tightly linked to HIV infectivity [25,27,44]. Cholesterol depletion significantly reduces HIV-1 particle production [29-34,44]. There is also a marked decrease in infectivity of virions produced from such cells [26]. The significant reduction correlates with the amount of virion-associated cholesterol [35]. In the current study, we clearly established that Cav-1 significantly reduces infection with virions produced from Cav-1 treated cells when compared to that of the same number of virions obtained from untreated cells. We have previously shown that Cav-1 represses HIV gene expression by blocking the NF-κB pathway thus subsequently affecting virus production [18]. The decrease in virus production is therefore in part due to transcriptional suppression of HIV gene expression. Here, we examined the cholesterol content of HIV particles produced from Cav-1 treated cells and clearly established a significant cholesterol decrease in virus particles. Furthermore, normalized amounts of virus in the infectivity assay of HIV released from Cav-1 treated cells shows that infectivity is markedly reduced. Normalized amounts of virus to assay for infectivity, rules out any concern regarding the level of virus release contributing to the reduction of infectivity. The major step that causes a decrease in virion infectivity related to cholesterol depletion is the fusion steps of infection [30]. In support of this notion, we previously demonstrated that Cav-1 significantly suppressed Env-induced membrane hemifusion [16], indicating that the decrease in fusion partly involves a reduction in the cholesterol composition of the plasma membrane. Cav-1 can counter the influence of HIV on cholesterol metabolism by promoting cholesterol trafficking to the membrane subsequently enhancing cholesterol efflux, therefore, depriving the HIV virion of cholesterol. Since Cav-1 is involved in cholesterol metabolism the up-regulation of Cav-1 can have an impact on the level of cellular cholesterol thereby contributing to a reduction in virus production and infectivity, consequently contributing to a persistent infection of macrophages.

## Conclusion

**Figure 8:**
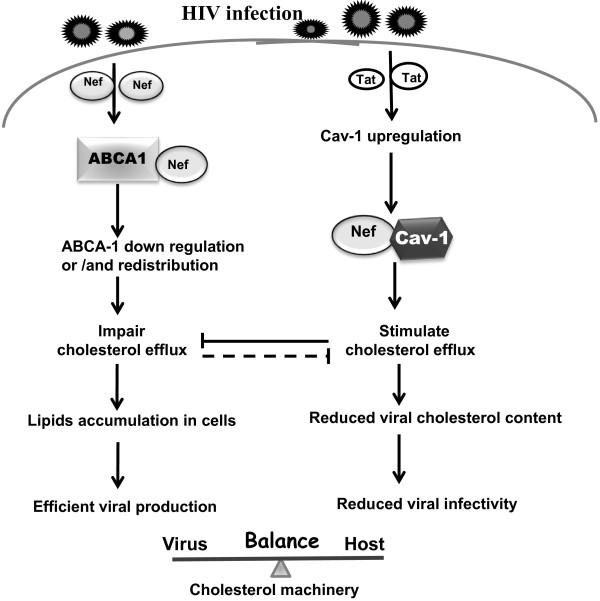
**A model for the interplay of Cav-1 with Nef that counters Nef induced impairment of cholesterol efflux by apoA-l and contribution to persistent infection.** Upon HIV infection, Nef protein interacts with ABCA-1 and down-regulates and/or redistributes ABCA-1 expression which results in cholesterol efflux impairment. This creates a micro-environment in which HIV can replicate efficiently. On the other hand, in cells which express Cav-1, such as macrophages, Tat induces an up-regulation of Cav-1. The overabundance of Cav-1 then leads to both its binding to Nef and ability to activate cholesterol efflux. This, therefore, leads to less cholesterol accumulation which in turn reduces the amount that can be incorporated into viral particles and thereby reducing HIV infectivity.

Infected macrophages are relatively resistant to cytopathic effect and consequently play an essential role in viral dissemination to host tissues and organs [53-55]. Furthermore, in this viral reservoir HIV infection appears not to be associated with apoptosis but with a chronic productively infected phenotype [56,57]. Although several mechanisms have been proposed we still don’t have a clear picture as to the mechanisms of persistent infection in macrophages. The relationship between HIV-1 and host factors determines the modulation of both cellular functions and virus replication within an infected individual, and with the interaction of these viral and cellular factors being evident in all steps of virus replication [58-62]. Their associations may be an important factor in the modification of host cell processes during a chronic viral infection. This suggests that a persistent infection is regulated by cellular factors at different steps in virus replication. Gene expression profiles that are unique to macrophages [63-65] when compared to that of activated CD4+ T cells should help determine the mechanism of persistent infection in macrophages. Cav-1 is highly expressed in terminally differentiated or quiescent cells including dendritic cells and monocytes/macrophages [2,66]. While macrophages express Cav-1, human T cells are generally believed to lack the Cav-1 protein [67-69]. The lack of Cav-1 in T cells, our discovery that HIV infection enhances Cav-1 expression mediated by Tat in macrophages and that Cav-1 reduces HIV replication [17] suggests a role for Cav-1 in an HIV persistent infection of macrophages. Here we have shown that Cav-1, by restoring cholesterol efflux impaired by Nef and subsequently influencing the cholesterol content of HIV particles which negatively affects virus infectivity, effectively inhibits HIV replication contributing to macrophage HIV persistent infection (Figure 8). 

## Methods

### Plasmids

The HIV-1 proviral constructs pNL4-3 (T-tropic), pNL-AD8 (M-tropic), pWT/Bal (M-tropic), pNL4-3.Luc.R-E-, and pSG3^Δenv^ were kindly provided by NIH AIDS Research and Reference Reagent Program [70-76]. The construct pNL4-3.Luc.R-E- is defective for env and nef where as pSG3^Δenv^ has intact nef, but a deletion in env. An expression plasmid for vesicular stomatitis virus envelope G protein (pCI-VSV) was kindly provided by Jiing-Kuan Yee of City of Hope National Medical Center, Duarte, California. A Cav-1 expressing plasmid, pCZ-cav-1, was generated as described previously [16]. pCZ-vector is the same as pCZ-cav-1 except it lacks the coding sequence of cav-1. The Nef expression plasmid pcDNA3.1SF2Nef was provided by NIH AIDS Research and Reference Reagent Program [77,78]. A construct expressing Nef tagged with HA (pCI NL4-3 Nef-HA-WT) was purchased from Addgene Inc (Cambridge, MA). The NefG2A mutation plasmid was generated using a site-directed mutagenesis kit according to the manufacturer’s protocol (Strategene). Briefly, the mutation was generated by PCR amplification using pCI NL4-3 Nef-HA-WT as template and the following pair of primers: 5^′^-ggattttgctataagatggctggcaagtggtcaaaaagt-3^′^ and 5^′^-actttttgaccacttgccagccatcttatagcaaaatcc-3^′^. The PCR products were digested with the restriction enzyme *DpnI* to destroy template plasmids and were then transformed into DH5α competent cells. Introduction of the mutation (pCI NL4-3 NefG2A-HA) was confirmed by sequence analysis. Wild type AD8 and a replication competent nef defective AD8 derived HIV provirus DNA construct ADnefmut [79] were provided by Dr. Maureen Goodenow of the University of Florida. Adenovirus particles (Ad) for expressing Cav-1 (Ad-Cav-1) and GFP (Ad-GFP) were obtained from Vector Biolabs (Philadelphia, PA).

### Cell cultures

Human U87MG-CD4 cells stably transfected with CXCR4 (U87-CD4-CXCR4) or CCR5 (U87-CD4-CCR5), human acute monocytic leukemia (THP-1),and an indicator cell line for tittering HIV (TZM-bl) was kindly provided by the NIH AIDS Research and Reference Reagent Program. U87-CD4-CXCR4 were maintained in DMEM containing 15% FBS, penicillin-streptomycin (100 μg/mL), glutamine, puromycin (1μg/ml; Sigma Chemical), and neomycin (G418; 300μg/ml; Sigma). THP-1 cells were grown in RPMI-1640 containing 10% FBS, 1.0mM sodium pyruvate, and 0.05 mM 2-mercaptoethanol. For differentiation into macrophages, THP-1 cells were treated with 50 ng/ml of phorbol 12-myristate 13-acetate (PMA, Sigma Chemical) for 5 days until the cells adhered and exhibited macrophage-like morphology. TZM-bl and 293T cells were grown in DMEM medium supplemented with 10% FBS and penicillin-streptomycin (100μg/ml). All cultures were maintained at 37°C in a humidified atmosphere with 5% CO2.

Peripheral blood mononuclear cells (PBMCs) were isolated from buffy coats prepared from healthy donors by centrifugation through a Ficoll gradient (Sigma-Aldrich, St. Louis, MO). Monocytes were isolated by negative selection with a human monocyte enrichment kit according to the manufacturer’s instructions (EasySep® Human Monocyte Enrichment Kit, Stemcell Technologies). The monocyte preparations contained 97% CD14^+^ cells, as determined by flow cytometry. For differentiation of monocytes into macrophages (MDMs), monocytes were seeded into Biocoat poly-D-lysine plates (B.D. Bioscience), and cultured in DMEM, supplemented with 10% heat-inactivated human serum, gentamicin (50μg/ml), ciprofloxacin (10μg/ml), and M-CSF (1000U/ml) for 7 days. MDM culture medium was half-exchanged every 2 to 3 days.

### Transfection of siRNA

Small interfering RNA (siRNA) targeting Cav-1 and control siRNA were purchased from Santa Cruz Biotechnology, Inc. Transfection of siRNA was performed using Oligofectamin^TM^ Reagent (Invitrogen Corp., Carlsbad, Calif.) according to the manufacturer’s protocol. Briefly, the day before transfection, U87cells were seeded into a 24 well plate and cultured with antibiotics free medium to 30% confluency. Cells were washed and resuspended in 200ul serum free medium. Transfection mixture was prepared by incubating 50pmol of siRNA duplexes with 3ul of Oligofectamin in a final volume of 50ul Opti-MEM I Medium. After a 5 hour incubation, 125ul of growth medium with 3 times the normal concentration of serum was added to cells. Transfection was repeated once the next day. For THP1 macrophages, cells were first transfected with siRNA followed by HIV infection, and cells were then transfected again with siRNA the day after infection. The efficiency of Cav-1 knock-down by the siRNA transfection was monitored using Western blot analysis.

### Virus production and concentration

Infectious virus HIV-1 AD8, ADnefmut, Bal, and NL4-3 were generated by calcium phosphate transfection of monolayers of 293T cells in 75-cm^2^ flasks with 25μg provirus DNA. Supernatants containing virus were harvested 4 days after transfection and quantified using the TZM-bl indicator cells as well as by measuring reverse transcriptase and a p24 ELISA method as described previously [17]. When required, virus was produced from U87-CD4-CXCR4 cells transfected with 18μg proviral HIV NL4-3 along with 9μg pCZ-Cav-1 or pCZ-vector. To generate pseudotyped HIV particles 20μg pSG3^Δenv^ or pNL4-3.Luc.R-E- was co-transfected with 3μg pCI-VSV into monolayers of 293T cells in 75-cm^2^ culture flasks by the calcium phosphate method. Pseudotyped viral supernatants were harvested 4 days post-transfection and were clarified by centrifugation at 3,000 rpm for 20 min and then by filtering through a 0.45 μm-pore size filter. Virus particles were concentrated using virus precipitation reagent Retro-Concentin^TM^ (System Biosciences) according to the manufacturer’s protocol.

### Oil red O staining

To determine the influence of Cav-1 on the level of lipid accumulation in HIV infected and uninfected cells oil red O staining was performed. THP-1 cells were differentiated into macrophages by treatment with 50 ng/ml PMA for 5 days then infected with HIV AD8 (moi, 0.01) or Bal (moi, 0.001). On day 10 post infection, cells were loaded with cholesterol by incubating with 50μg/ml Ac-LDL (Biomedical Technologies Inc., Stoughton, MA) for 48 h followed by 30μg/ml apoA-I stimulation for 18 hours. Differentiated THP-1 cell-differentiated macrophage cells were also infected with Ad-Cav-1 or Ad-GFP at an moi (multiplicity of infection) of 100. Twenty-four hours later they were infected with VSV pseudotyped HIV pSG3^Δenv^ or pNL4-3.Luc.R-E- at an moi of 3 and incubated for 5 days. Oil red O staining was performed as previously described [80]. Briefly, cells were rinsed with PBS, followed by fixation with 3.7% paraformaldehyde for 60 min. The cells were stained using freshly prepared Oil red O (Sigma) working solution at room temperature for 10min. Intensity of cell staining was observed using a light microscope.

### Virus infectivity assay

To test Cav-1’s influence on HIV-1 infectivity, TZM-bl cells were infected with virus harvested from Cav-1 treated cells and the infectivity levels were measured by luciferase activity. MDMs were first infected with adenovirus expressing Cav-1 or GFP at an moi of 100 in serum free medium for 6 hours. The cells were then washed and incubated in serum-containing medium over-night, after which cells were infected with HIV AD8 at an moi of 0.1 for 6 hours, at which point they were washed and refreshed with new medium. On day 6 post infection, supernatants were subjected to RT assay or titered using the indicator TZM-bl cell line. Virus amounts were normalized with level of infectivity being assayed by measuring luciferase within TZM-bl cells [74]. Normalized amounts of virus were used for subsequent infections.

### Determination of cholesterol content and cholesterol replenishment assay

Equivalent amounts of virions were quantified by p24 assay and tested for cholesterol content using the Amplex Red cholesterol Assay Kit (Invitrogen, Carlsbad, CA) according to the manufacturer’s protocol. To replenish cholesterol virus amounts were also normalized by p24 assay and incubated in 0.5mM (2-Hydroxypropyl)-ß-Cyclodextrin solution (Sigma Aldrich) with 1.5mM cholesterol (Sigma Aldrich) at 37°C for 1 hour. These quantified and normalized amounts of virus were used to infect TZM-bl cells and monitored for luciferase activity.

### Cholesterol efflux

U87 cells were transfected with pcDNA3.1 and pCZ-vector (mock), pcDNA3.1SF2Nef and pCZ-vector (Nef), pCZ-cav-1 and pcDNA3.1 (Cav-1), pcDNA3.1SF2Nef and pCZ-cav-1 (Nef plus Cav-1), pCI NL4-3 NefG2A-HA (NefG2A) and pCZ-vector, or pCI NL4-3 NefG2A-HA and pCZ-cav-1 (NefG2A plus Cav-1). Twenty-four hours after transfection cell culture medium was replaced with serum free medium containing 2 μCi/mL [^3^H] cholesterol and 1.5% BSA and incubated for 36 hours. Radioisotope-containing medium was then removed and cells were washed twice with PBS and cultured for an additional 18 hours in serum free medium in the presence or absence of 50 μg/ml ApoA-l (Biomedical Technologies Inc., Stoughton, MA). Cholesterol content was measured in the cell free media as well as within cells after lysing using 0.1N NaOH. ApoA-l specific cholesterol efflux was determined using the formula: apoA-l specific efflux = % cholesterol efflux with apoA-l - % cholesterol efflux without apoA-l (blank); cholesterol efflux= [cpm(supernatants)/cpm(supernatants+cells)] ×100%. HDL mediated cholesterol efflux is also examined by incubating cells for 18 hours in the presence or absence of 50 μg/ml HDL (Biomedical Technologies Inc., Stoughton, MA).

To determine cholesterol efflux from macrophages, MDMs were first infected with Ad-Cav-1 or Ad-GFP at an moi of 50 for 24 hours, which was followed by infection of pseudotyped HIV pSG3^Δenv^ (psHIVwtNef) or pNL4-3.Luc.R-E- (psHIVΔNef). Five days post infection cells were then labeled with 1 μCi/mL [^3^H] cholesterol for 48 hours and apoA-l mediated cholesterol efflux was determined as described above. Similarly cholesterol efflux from THP-1 cell-differentiated macrophages was determined 21 days after infecting with HIV AD8 at an moi of 0.001. Cholesterol efflux was also determined 14 days after THP-1 cell-differentiated macrophages infected with an moi of 0.001, 0.01, or 0.1. In addition, primary macrophages (MDMs) were infected with AD8 or ADnefmut HIV with an moi 0.01 and then cultured cells were subjected to cholesterol efflux assay 15 days after infection. ABCA-1 expression was determined by Western blots in MDMs 14 days after co-infection with AD8 or ADnefmut HIV and with Ad-Cav-1 or Ad-GFP. Inhibition of HIV replication was performed by treating infected cells with 5 uM azidothymidine (AZT) (Sigma-Aldrich, St. Louis, MO).

### Immunoprecipitation and Immunoblotting analyses

U87 cells were transfected with pCZ-Cav-1 and HA-tagged Nef (pCI NL4-3 Nef-HA-WT) or HA-tagged NefG2A (pCI NL4-3 NefG2A-HA), followed by incubation of medium containing cholesterol (30μg/ml) for 48 hours. Cells were then treated with apoA-I (20μg/ml) for 30 min. Cells were put on ice, washed twice with cold PBS and total cellular protein was extracted in lysis buffer (50 mM Tris pH 7.5,100 mM NaCl, 1 mM EDTA, 0.1% (v/v) Triton X-100, 10 mM NaF, 1 mM phenylmethyl sulfonyl fluoride, and 1 mmol/L vanadate) with a complete protease Inhibitor mixture (Roche Diagnostics, Indianapolis, IN). The concentration of extracted protein was determined and adjusted to 1 ug/ul. A total of 500 ul was used for each immunoprecipitation, to which 2μg of antibodies (anti-Cav-1 or anti-HA) or normal IgG were added. The mixtures were incubated at 4°C overnight. Following the overnight incubation, 25 μl of protein A/G-agarose beads (Santa Cruz Biotechnology, Santa Cruz, CA) were added and the mixtures were then rotated for 2 hours at 4°C. The beads were harvested by centrifugation and washed five times with lysis buffer. Loading buffer was added and boiled for 5 min. The samples were subjected to SDS-PAGE and analyzed by immunoblotting as described previously [81]. The primary antibodies used for immunoblotting were rabbit polyclonal anti-Cav-1(Santa Cruz Biotechnology, Santa Cruz, CA), mouse monoclonal anti-Nef, rabbit Nef antiserum, and human monoclonal anti-Gag (NIH AIDS Research and Reference Reagent Program), goat polyclonal anti-HA (Genescript), mouse monoclonal anti-ABCA1 (abcam), and ß-actin protein antibody (Sigma, St. Louis, MO). The secondary antibodies were HRP-linked anti-rabbit, anti-mouse (Cell Signaling Technology, Inc., Danvers, MA), anti-human IgG (Sigma, St. Louis, MO) or anti-goat IgG (Santa Cruz Biotechnology, Santa Cruz, CA).

### Statistical analysis

Student’s t test was applied to analyze the differences between sets of data. All analyses were performed with SPSS 12.0.1 for Windows, and were considered significant at p ≤ 0.05.

## Competing interests

The authors declare that they have no competing interests.

## Authors’ contributions

SL Participated in the design of experiments, carried out most of the experiment, prepared samples and conducted cholesterol analysis and Western blots, analyzed data and contributed to manuscript preparation. PN participated and assisted in sample collections and experiments. XW was involved in the design of constructs used for the study as well as establish efficient methodology to deliver of protein of interest in primary macrophages. AM conceived the study, designed and coordinated experiments, participated in data analysis and prepared contributed the manuscript. All authors read and approved the final manuscript.
